# Supplementary material for: Resolving the full spectrum of human genome variation using Linked-Reads
Source: Genome Res. 2019 Apr;29(4):635–45. doi: 10.1101/gr.234443.118 (PMC6442396; doi:10.1101/gr.234443.118)
Supplement: Supplemental Material [file supp_gr.234443.118_Supplemental_longranger-2.2.zip › longranger-2.2.tar.bz2!/lr2.2-public/lib/stan/cmdstan/stan/doc/api/html/csv__writer_8hpp_source.html]

Stan: src/stan/io/csv\_writer.hpp Source File


|  |  |
| --- | --- |
|  | Stan  2.5.0  probability, sampling & optimization |


- Main Page
- Related Pages
- Namespaces
- Classes
- Files

- File List
- File Members

All Classes Namespaces Files Functions Variables Typedefs Enumerations Enumerator Friends Macros Pages

- src
- stan
- io

csv\_writer.hpp

Go to the documentation of this file.

1 #ifndef STAN\_\_IO\_\_CSV\_WRITER\_HPP

2 #define STAN\_\_IO\_\_CSV\_WRITER\_HPP

3

4 #include <ostream>

5 #include <limits>

6 #include <iomanip>

7 #include <stan/math/matrix.hpp>

8 #include <stan/math/meta/index\_type.hpp>

9 #include <stan/math/matrix/meta/index\_type.hpp>

10

11 namespace stan {

12

13  namespace io {

14

23  class csv\_writer {

24  private:

25  std::ostream& o\_;

26  bool at\_bol\_;

27

28  public:

29

36  void comma() {

37  if (at\_bol\_) {

38  at\_bol\_ = false;

39  return;

40  }

41  o\_ << ",";

42  }

43

44

51  csv\_writer(std::ostream& o)

52  : o\_(o), at\_bol\_(true) {

53  }

54

63  void newline() {

64  o\_ << "\n";

65  at\_bol\_ = true;

66  }

67

76  void write(int n) {

77  comma();

78  o\_ << n;

79  }

80

89  void write(double x) {

90  comma();

91  o\_ << std::setprecision (std::numeric\_limits<double>::digits10 + 1)

92  << x;

93  }

94

103  template<int R, int C>

104  void write\_row\_major(const Eigen::Matrix<double,R,C>& m) {

105  using Eigen::Matrix;

106  using stan::math::index\_type;

107  typedef typename index\_type<Matrix<double,R,C> >::type idx\_t;

108  for (idx\_t i = 0; i < m.rows(); ++i)

109  for (idx\_t j = 0; j < m.cols(); ++j)

110  write(m(i,j));

111  }

112

121  template<int R, int C>

122  void write\_col\_major(const Eigen::Matrix<double,R,C>& m) {

123  using Eigen::Matrix;

124  using stan::math::index\_type;

125  typedef typename index\_type<Matrix<double,R,C> >::type idx\_t;

126

127  for (idx\_t j = 0; j < m.cols(); ++j)

128  for (idx\_t i = 0; i < m.rows(); ++i)

129  write(m(i,j));

130  }

131

132  template<int R, int C>

133  void write(const Eigen::Matrix<double,R,C>& m) {

134  write\_col\_major(m);

135  }

136

146  void write(const std::string& s) {

147  comma();

148

149  o\_ << '"';

150  for (size\_t i = 0; i < s.size(); ++i) {

151  if (s.at(i) == '"') {

152  o\_ << '"' << '"'; // double quotes

153  } else {

154  o\_ << s.at(i);

155  }

156  }

157  o\_ << '"';

158  }

159

160

161  };

162

163  }

164

165 }

166

167 #endif

stan::io::csv\_writer::newline

void newline()

Write a newline character.

**Definition:** csv\_writer.hpp:63

stan::io::csv\_writer::write\_col\_major

void write\_col\_major(const Eigen::Matrix< double, R, C > &m)

Write a value in column-major order.

**Definition:** csv\_writer.hpp:122

stan::io::csv\_writer::write

void write(const std::string &s)

Write a value.

**Definition:** csv\_writer.hpp:146

stan::math::index\_type

Primary template class for the metaprogram to compute the index type of a container.

**Definition:** index\_type.hpp:21

stan::io::csv\_writer

Writes Stan variables in comma-separated-value format to an output stream.

**Definition:** csv\_writer.hpp:23

stan::io::csv\_writer::write

void write(int n)

Write a value.

**Definition:** csv\_writer.hpp:76

stan::io::csv\_writer::csv\_writer

csv\_writer(std::ostream &o)

Construct a CSV writer that writes to the specified output stream.

**Definition:** csv\_writer.hpp:51

index\_type.hpp

stan::io::csv\_writer::comma

void comma()

Write a comma.

**Definition:** csv\_writer.hpp:36

stan::io::csv\_writer::write\_row\_major

void write\_row\_major(const Eigen::Matrix< double, R, C > &m)

Write a value.

**Definition:** csv\_writer.hpp:104

stan::io::csv\_writer::write

void write(double x)

Write a value.

**Definition:** csv\_writer.hpp:89

stan::io::csv\_writer::write

void write(const Eigen::Matrix< double, R, C > &m)

**Definition:** csv\_writer.hpp:133

index\_type.hpp

matrix.hpp


---

[ Stan Home Page ]

*© 2011–2014,
Stan Development Team.*
